# Treating Diphenhydramine Overdose: A Literature Review of Currently Available Treatment Methods

**DOI:** 10.3390/toxics12060376

**Published:** 2024-05-21

**Authors:** Jayna Patel, Joshua Edwards

**Affiliations:** 1 Chicago College of Osteopathic Medicine, Midwestern University, 555 31st Street, Downers Grove, IL 60515, USA; 2 College of Graduate Studies, Midwestern University, 555 31st Street, Downers Grove, IL 60515, USA

**Keywords:** drug toxicity, antihistamine, physostigmine, sodium bicarbonate, donepezil, dexmedetomidine, lipid emulsion therapy

## Abstract

From 2019 to 2020, antihistamines were found in 15% of all US drug overdose deaths, often co-administered with fentanyl, with 3.6% of overdose deaths due to antihistamines alone. The most common antihistamine found in all these reported deaths is diphenhydramine, a ubiquitous, over-the-counter and clinically important medication. Currently, there is no antidote for diphenhydramine overdose. This review summarizes the adverse health effects and current emergency medicine treatments for diphenhydramine. Several emergency medicine case reports are reviewed, and the efficacy and outcomes of a variety of treatments are compared. The treatments reviewed include the more traditional antihistamine overdose therapeutics physostigmine and sodium bicarbonate, as well as newer ones such as donepezil, dexmedetomidine, and lipid emulsion therapy. We conclude that more study is needed to determine the ideal therapeutic approach to treating antihistamine overdoses.

## 1. Background

### 1.1. Diphenhydramine

Diphenhydramine is an extremely common antihistamine that has been used for almost 80 years to treat a wide variety of conditions in most age groups it is often used for the treatment of seasonal allergies and sleeping difficulties [1]. For many years, diphenhydramine was used to sedate neonates who had difficulty sleeping; however, the TIRED study found no statistically significant benefit, and diphenhydramine is now contraindicated in neonates and is only used with caution in infants and young children [2]. Commonly known as Benadryl [1], diphenhydramine is also present in many other over-the-counter medications [3]. Due to its long history on the market, many patients and healthcare professionals are closely familiar with diphenhydramine, which contributes to the drug’s continued popularity in treatment [1].

Diphenhydramine is a first-generation H1-antihistamine that functions to reduce allergic responses as a competitive antagonist in opposing the binding of histamine at H1-receptors and as inverse agonists, causing reduced baseline activity of the H1-receptor. This class of drugs is often used to treat many different allergic and nonallergic conditions, including cough, acute otitis media, upper respiratory tract infection, sinusitis, nonallergic itching and via actions in the central nervous system (CNS) that can cause sedation and treat motion sickness. Second-generation antihistamines do not cross the blood–brain barrier and therefore have fewer CNS effects. First-generation H1-antihistamines are able to bind to H1 receptors on neurons in the CNS. In addition, first generation H1-antihistamines are not highly selective for the H1-receptor, and they can also bind other types of receptors, such as muscarinic and human bitter taste receptors [4]. Diphenhydramine may even alter voltage-gated sodium channels during the propagation of action potentials with high affinity (Kd = 10 µM) for sodium channels in the inactive state [5]. This combination of characteristics means that first-generation H1-antihistamines often cause multiple and severe side effects due to their direct actions on the autonomic and somatic CNS in addition to the vascular system [6].

### 1.2. Diphenhydramine Risks

When diphenhydramine was first introduced in 1946, there were not very high standards for drug testing and safety [1]. Thus, first generation antihistamines were released without approval from regulatory bodies and without sufficient clinical testing [6]. As such, diphenhydramine’s potential for causing adverse health effects was not appropriately recognized for many years. In addition, diphenhydramine may not even effectively treat many of the conditions for which it has been historically applied. Examples of such conditions for which diphenhydramine treatment may not in fact be sufficient include anaphylaxis, atopic dermatitis, nonallergic angioedema, otitis media, upper respiratory tract infections, sinusitis, nonallergic itching, and cough [6]. 

Due to its potential to cross the blood–brain barrier to cause CNS effects, the FDA has issued a warning that overdosing on diphenhydramine may cause seizures, coma, and even death [7]. The volume of distribution for diphenhydramine is 292–480 L, indicating much of the drug dose becomes redistributed outside the blood [8]. Commonly, diphenhydramine can result in sedation in patients at therapeutic doses. This is a result of its ability to cross the blood–brain barrier and also to induce its anticholinergic function at various types of receptors in the body [1]. In addition, histamine may be crucial to the circadian rhythm, particularly in the process of waking [9]; this may also explain diphenhydramine’s potential for sedation due to its antihistamine properties. Thus, diphenhydramine is frequently used therapeutically in treating insomnia, inducing analgesia and conscious sedation, and in managing headaches, anxiety, and agitation [6]. Additionally, diphenhydramine’s wide range of action allows it to act on cardiac ion channels, including sodium channels and even potassium channels [10]. This can cause adverse cardiac effects such as QRS widening and lengthening of the QT interval [10]. Diphenhydramine can have activity at alpha-adrenergic receptors, which may lead to dizziness and orthostatic hypotension [1]. Other side effects of diphenhydramine can include antimuscarinic symptoms like dry mouth, constipation, mydriasis, and urinary retention, as well as antiserotonin symptoms like increased appetite [6]. Various side effects and a lack of standard testing associated with diphenhydramine have placed the drug under increased scrutiny for current use. Some authors have begun arguing for a complete shift away from first-generation antihistamines altogether, favoring second-generation antihistamines, which are more selective for the H1 receptor and thus have few side effects, or other classes of drugs [6]. 

### 1.3. Diphenhydramine Overdoses

**Figure 1:**
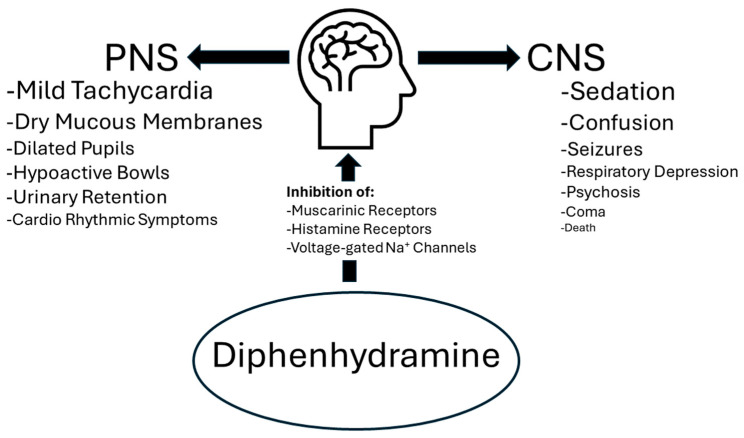
Summary figure showing target receptors and effects of diphenhydramine overdose in the peripheral nervous system (PNS) and central nervous system (CNS). The more common the toxicological effect listed, the larger the relative font size.

Given that diphenhydramine has been revealed to be riskier than expected, even at safe doses, diphenhydramine taken in overdose can even further endanger patients’ health. Diphenhydramine overdose typically results in a presentation of anticholinergic toxicity that can manifest both in central and peripheral nervous symptoms, the severity of which depends on the dose [11], see Figure 1. In mild oral overdoses of less than 300 mg [11], the patient usually presents with symptoms including tachycardia, dry mucous membranes, dilated pupils, hypoactive bowel sounds, and urinary retention [12]. Higher doses can also result in agitation and hallucinations [12]. In doses greater than 1 g, symptoms may also include seizures, delirium, psychosis, rhabdomyolysis, coma, and death [11]. Extreme overdoses can elicit adverse cardiorhythmic symptoms as well [11,12]. Other symptoms often observed in diphenhydramine overdoses include confusion, drowsiness, and respiratory depression [6]. Adults most commonly present with sedation and coma while children may be more likely to also experience agitation [1], hallucinations, and seizures [6]. 

Moreover, diphenhydramine overdose levels have been rising, making this an extremely pertinent public health issue, including the popular Tik Tok “Benadryl Challenge”, where children and teenagers intentionally overdose on OTC Benadryl and record and post their altered behavior [7]. United States poison control centers have extremely often documented diphenhydramine overdose cases. From 2019 to 2020, 15% of all drug overdose deaths in the US were anti-histamine positive, while 3.6% of overdose deaths were antihistamine-involved, often co-administered with fentanyl. Of the anti-histamines, diphenhydramine is by far the most common, with over 71% of drug overdose deaths [13]. 

## 2. Treatments

In order to manage rising diphenhydramine overdose rates, effective treatments are needed more than ever. Healthcare professionals should familiarize themselves with the available treatment methods for diphenhydramine overdose. Currently, in lieu of a specific antidote for diphenhydramine toxicity, standard treatment for diphenhydramine overdose cases has remained supportive care [9,10,11,14,15], with the administration of benzodiazepines for seizures and agitation [10,11], and sometimes hemodialysis when needed [15]. However, benzodiazepines poorly manage agitation symptoms in most patients, and can even cause adverse effects such as respiratory depression and increased delirium [10]. Thus, current standards of treatment for diphenhydramine overdose are often insufficient and can even be dangerous. Although research into effective diphenhydramine toxicity therapies has been limited, recent years have elucidated several promising alternate methods of treatment for diphenhydramine overdose: physostigmine, sodium bicarbonate, donepezil, dexmedetomidine, and lipid emulsion therapy.

### 2.1. Physostigmine

Physostigmine is an acetylcholinesterase inhibitor that can travel into the CNS. Thus, it can act at both central and peripheral synapses to inhibit acetylcholinesterase and increase acetylcholine levels [16]. This mechanism of action could be particularly useful in opposing the anticholinergic effects at muscarinic receptors caused by diphenhydramine toxicity. 

Several studies have investigated the therapeutic potential of physostigmine. In a 2014 report by Phillips et al., a case is described in which a 13-year-old female intentionally overdosed on various pills for self-harm purposes. The exact dose was unknown. The patient presented with a lack of sweating, dilated pupils, dry mucous membranes, as well as fever, agitation, myoclonus, rigidity, hallucinations, tachypnea, and tachycardia. After 7 mg of lorazepam was given with little change in symptoms, the patient was started on intravenous physostigmine treatment. She received two 2-mg doses over the space of 2 h with slight improvement after each dose, then received a continuous infusion for 8 h and another 2 mg dose, during which the patient’s antimuscarinic symptoms improved and after which hallucinations and agitation also resolved. Additionally, benzodiazepines and phenobarbital were given to resolve her myoclonus and rigidity. The authors concluded that physostigmine can effectively treat antimuscarinic symptoms of diphenhydramine overdose [17]. Similarly, a 2002 study by Padilla and Pollack describes the case of a 31-year-old male patient who had been ingesting approximately 750 mg diphenhydramine each day for multiple months; on the day of presentation, the patient had altered mental status and was involved in a motor vehicle accident. In addition to altered mental status and a lack of meaningful speech, the patient was agitated, hypertensive, and tachycardic, with dry skin and mucous membranes. The patient received several doses of intravenous physostigmine and had normal vital signs by 6 h after the final physostigmine dose [14].

The findings of these reports are backed by a 2000 retrospective study comparing the efficacy of physostigmine to benzodiazepines in 52 cases of anticholinergic toxicity. Physostigmine has historically been thought to be dangerous due to some reports of its causing adverse cardiac side effects, such as asystole. However, in their study, Burns and colleagues found that physostigmine resolved symptoms of agitation in 96% of patients, whereas benzodiazepines only managed these symptoms in 24% of patients. Physostigmine also resolved delirium in 87% of patients, while benzodiazepines did not resolve delirium in any patients. Although physostigmine and benzodiazepines resulted in statistically equal adverse side effects, the side effects caused by physostigmine resolved without any treatment, whereas benzodiazepines caused further sedative side effects that lengthened symptoms for 2 patients [16]. Existing research suggests that the use of physostigmine may be favorable to the supportive care and benzodiazepines that are standardly administered in cases of diphenhydramine overdose. Further investigations are needed to fully elucidate the safety and treatment potential of physostigmine. As mentioned previously, there may be a potential for adverse cardiac effects that may limit use of the drug. Another concern with physostigmine and other acetylcholine esterase inhibitors is being able to give the correct dose without overdosing, as these drugs have indirect effects on increasing the concentrations of the ligand for muscarinic receptors, acetylcholine.

### 2.2. Sodium Bicarbonate

As well as acting as an antihistamine and an anticholinergic, diphenhydramine also blocks type IA sodium channels [5]. Thus, diphenhydramine overdose can cause cardiac symptoms in addition to the typically seen anticholinergic toxicity [3]. The toxic blockage of type IA sodium channels can lead to various adverse cardiac effects, such as QRS widening [18]. Historically, sodium bicarbonate has been widely used to combat the blockage of type IA sodium channels by various drugs, most notably tricyclic antidepressants [3,18]. Administering sodium bicarbonate increases extracellular sodium levels, thus opposing the blockage [19]. Due to this history of success, several authors have sought to investigate the efficacy of sodium bicarbonate in treating diphenhydramine toxicity. 

A 2003 study by Sharma and colleagues describes three cases in which sodium bicarbonate was used to treat diphenhydramine overdose. In one case, a 23-year-old female patient ingested 90 tablets of diphenhydramine and soon began to develop toxic symptoms, including high blood pressure, tachycardia, and a long QRS interval of 128 msec, as well as multiple tonic–clonic seizures. Despite lorazepam and phenobarbital being administered and the seizures resolving with the administration of midazolam and vecuronium, the patient’s toxic symptoms continued. Finally, the patient was treated with 50 mL of 89.2 mEq/L hypertonic intravenous sodium bicarbonate. Within thirty minutes, her QRS interval shortened to 89 msec, and within the next few days, all other toxic symptoms resolved, allowing for her discharge. In another case, a 39-year-old male patient presented with a diphenhydramine overdose. The exact dosage was unknown, but he experienced overdose symptoms including dilated pupils, tachycardia, and a long QRS interval of 162 msec, in addition to a tonic–clonic seizure followed by unresponsiveness. The patient eventually died 10 h after hospital admission, but during his treatment, the administration of sodium bicarbonate caused his QRS interval to shorten to 151 msec. In the third case, a 41-year-old female patient ingested 150 Excedrin PM tablets; the total possible dosage was calculated to be 57.75 g diphenhydramine, along with 75 g acetaminophen. The patient had high blood pressure and slight tachycardia, which eventually progressed to a lengthened QRS interval of 102 msec, despite initial treatment with activated charcoal, sorbitol, and N-acetylcysteine. After being given 25 mL of 44.6 mEq intravenous sodium bicarbonate, her QRS interval shortened to 86 msec. She continued to improve, and she was discharged two days later [3]. Taken together, these three cases indicate that sodium bicarbonate could be an especially useful treatment for severe diphenhydramine overdoses, where cardiac symptoms including QRS widening may be involved. 

A 2011 study by Cole and colleagues demonstrates that similar benefits of sodium bicarbonate can be seen in cases of pediatric diphenhydramine overdose. In the study, a case is described of a 13-month-old female patient who ingested 24 tablets of 25 mg diphenhydramine each, for a maximum possible dosage of 50 mg/kg. She presented with tonic–clonic seizure, which resolved with midazolam, as well as altered mental status, tachycardia, a long QRS interval of 130 msec, and a long QTc interval of 506 msec. After being treated with a 1 mEq/kg dose of hypertonic sodium bicarbonate, within 98 min, the QRS interval shortened to 66 msec and the QTc shortened to 482 msec. By the next day, all other symptoms had resolved, allowing for her discharge [19]. Although more studies are needed to solidify the current understanding of sodium bicarbonate’s effects in the body and in various demographics, existing literature suggests that sodium bicarbonate could effectively be used to manage the adverse cardiac symptoms of severe diphenhydramine overdose cases. 

### 2.3. Donepezil

Donepezil hydrochloride is a piperidine-based drug that functions as a cholinesterase inhibitor [20,21]. Originally, it was approved as a treatment to augment cholinergic activity in Alzheimer’s disease, which is characterized by a reduced cholinergic system in the brain [21]. Donepezil HCl was designed to specifically target the acetylcholinesterases of the CNS, leaving the cholinesterases of the plasma largely untouched [20,21]. 

Research has elucidated the pharmacokinetic characteristics of donepezil that lend it unique therapeutic potential. In 48 healthy adult males, donepezil inhibited nearly 35% of peripheral erythrocyte acetylcholine esterase and had a relatively long half-life of ~ 80 hrs with few adverse effects [21]. In addition, donepezil is highly selective for brain acetylcholine esterase, with a 1252-fold higher ability to inhibit acetylcholine esterase from butyrylcholine esterase in plasma [22]. 

Donepezil’s properties as a cholinesterase inhibitor highly specific for the CNS make it a well-suited candidate for possible use in treating diphenhydramine overdoses. In a 2019 study by Ahmad and colleagues, a case is described of an 89-year-old male patient in Bangladesh who ingested 80 tablets of 50 mg diphenhydramine each, for a total of 4 g ingested diphenhydramine. He presented with unconsciousness as well as tachycardia, tachypnea, high blood pressure, dry mouth, urinary retention, and a lack of bowel sounds. Along with mechanical ventilation and a urinary catheter, the patient was given donepezil HCl, which was chosen as an alternative to physostigmine since this drug was unavailable. He received a total of 10 mg of enteral donepezil each day for 5 days. The patient eventually died, but initial improvements in his condition were seen after donepezil administration. On the first day of donepezil treatment, the patient’s Glasgow Coma Scale score improved, he was extubated, his anticholinergic symptoms reduced, and by the sixth day, he was discharged from the ICU. He died 12 days later from a myocardial infarction [20].

More research is needed to better elucidate the therapeutic potential of donepezil HCl in diphenhydramine overdose. However, current understandings of donepezil support its use in treating the anticholinergic symptoms of diphenhydramine toxicity, while avoiding additional peripheral side effects that may arise in other treatment methods for diphenhydramine overdose. In fact, donepezil has been found to have higher selectivity for acetylcholinesterases in brain tissue than physostigmine [22]. Further studies should continue to investigate the efficacy and safety of donepezil HCl when compared to other treatments for diphenhydramine overdose.

### 2.4. Dexmedetomidine

Dexmedetomidine is a selective agonist for alpha-2 adrenergic receptors [10,11]. It mainly acts on receptors in the medulla, causing a reduction in sympathetic activity at the locus coeruleus in the brain and thereby resulting in the increased activity of inhibitory GABA neurons [11]. Thus, dexmedetomidine achieves sympatholytic effects, which allow many therapeutic uses, including sedation [10,11] and analgesia [11]. Despite this mechanism of action, dexmedetomidine has only very limited effects on respiration [10,11]. Historically, this combination of properties has made dexmedetomidine an ideal candidate for treating alcohol withdrawal syndrome, a condition characterized by excessive sympathetic activity resulting in symptoms such as tachycardia, agitation, high blood pressure, hallucinations, tremors, and seizures. Given that these symptoms are similar to those experienced by patients in cases of anticholinergic toxicity, it has been thought that dexmedetomidine could be used to treat such cases as well [11]. 

Several studies have sought to explore this therapeutic potential in cases of diphenhydramine overdose. In a 2014 study by Walker and colleagues, a case is described of a 13-year-old female patient who ingested 24 tablets of 25 mg diphenhydramine each for a total ingestion of 600 mg diphenhydramine, 9.5 mg/kg. She presented with tachycardia, high blood pressure, incoherent speech, dilated pupils, and dry mucous membranes. Despite receiving lorazepam, her condition continued to worsen, and she soon began experiencing tachypnea, increased agitation, and visual hallucinations. Finally, intravenous dexmedetomidine was administered, first as a 1 mcg/kg loading dose and then as a 0.5 mcg/kg/h infusion, which was maintained for approximately 25 h. The patient’s agitation and tachycardia immediately improved. When the infusion was removed at 25 h, the patient began experiencing hallucinations again, so the infusion was started again for another 8 h. After this, the patient’s symptoms had completely resolved, apart from some feelings of anxiety afterwards, which were managed using lorazepam [11].

A 2021 study by Zekhtser and colleagues details two polydrug overdose cases in which dexmedetomidine was therapeutically used to treat anticholinergic toxicity. In one case, a 2-year-old male patient is described who ingested an unknown amount of Dimetapp; the contents were also unknown, as available Dimetapp variations can include diphenhydramine as well as other drugs such as phenylephrine, brompheniramine, and dextromethorphan. The patient presented with typical symptoms of anticholinergic toxicity, including tachycardia, high blood pressure, and dry mucous membranes, in addition to other symptoms such as hypopnea and lethargy, which may suggest a poly ingestion. A dexmedetomidine infusion was given for the first hour at 0.3 mcg/kg/h, then afterwards at 0.2 mcg/kg/h for one more hour. After this treatment, his tachycardia and high blood pressure resolved quickly, and all other symptoms gradually resolved as well, and he was soon discharged. In this case, due to the potential for benzodiazepines to worsen respiratory depression, dexmedetomidine was instead chosen for administration in light of the patient’s hypopnea. The authors stress the possible unique safety of dexmedetomidine in cases such as these where patients may ingest multiple drugs and treatment must improve anticholinergic symptoms without worsening other types of symptoms. This is further supported by the second case, in which a 14-year-old female patient ingested more than 100 tablets of 25 mg diphenhydramine each, and likely ingested multiple other over-the-counter drugs as well; her labs also indicated the presence of cannabinoids and acetaminophen. She presented with tachycardia, a lengthened QTc interval, high blood pressure, agitation, hallucinations, dry skin, dilated pupils, and nonsensical speech, in addition to emesis and urinary incontinence. The patient received several doses of lorazepam, but symptoms continued. She was given dexmedetomidine at 0.3 mcg/kg/h which was titrated to 0.7 mcg/kg/h. As this titration occurred, her anticholinergic symptoms gradually resolved. By 13 h later, all symptoms had resolved, and she was discharged. Again, the authors emphasize the potential utility of dexmedetomidine in cases of anticholinergic toxicity where multiple drugs may be involved and where standard treatments like benzodiazepines are ineffective or risky [10]. 

The current literature seems to support the possible success of dexmedetomidine treatment in cases of diphenhydramine overdose. However, further research is needed to solidify our understanding of dexmedetomidine treatment in cases of diphenhydramine overdose alone and in cases of adult diphenhydramine overdose. Additionally, the limitations of dexmedetomidine should be considered. For instance, the antidote for dexmedetomidine is atropine, an anticholinergic that could exacerbate an existing anticholinergic toxicity; thus, dexmedetomidine should be administered with caution [10]. Dexmedetomidine can occasionally cause adverse side effects, including bradycardia and hypotension [11], although it has been argued that these effects are easily preventable by tightly controlling the dose amount [10]. Dexmedetomidine may be rendered ineffective when used for longer than 24 h due to tolerance and tachyphylaxis and may even cause withdrawal in some patients, although this may not be as severe in children [11]. On the other hand, dexmedetomidine can also lend unique benefits; it has a limited influence on respiration and on neurologic status [11]. It has a minimal effect on cardiac rhythms and on QTc interval, especially when compared with physostigmine. Dexmedetomidine may even help in reducing symptoms of delirium [10]. This particular profile of characteristics could make dexmedetomidine a useful treatment option to consider in overdose cases where diphenhydramine is ingested, and possibly even in cases where other drugs are also involved. Future studies should further explore the safety and benefits of dexmedetomidine treatment. 

### 2.5. Lipid Emulsion Therapy

Intravenous lipid emulsion therapy is a treatment that takes advantage of the lipophilic nature of some drugs in order to nullify those drugs [12,15]. In this treatment method, lipids in the emulsion bind with the lipophilic drug, thus redistributing the drug and reducing the possibility of the drug interacting with receptors [12]. Diphenhydramine happens to be lipophilic, making it a potential candidate for this treatment method [15]. As discussed previously, severe diphenhydramine toxicity can result in adverse cardiac effects due to the drug’s blockage of sodium channels [3]. Although we have already reviewed the uses of sodium bicarbonate in tackling these effects, some authors have argued that lipid emulsion therapy may be a valuable alternate treatment method to consider in such cases. In lipid emulsion therapy, the lipids of the emulsion would bind to diphenhydramine molecules, thus freeing sodium channels of the diphenhydramine blockage [15,23]. 

In a 2014 study, Abdi and colleagues report on the case of a 23-year-old male patient who ingested 2000–2500 mg of diphenhydramine. He presented with tachycardia, dilated and sluggish pupils, an absent corneal reflex, an absent response to pain stimuli, and dry skin. Importantly, he had a lengthened QRS of 172 msec and a lengthened QTc of 577 msec; he also had a tonic–clonic seizure. The patient was given midazolam for the seizure, then received 500 mEq sodium bicarbonate and began on a bicarbonate infusion, without improvement in anticholinergic symptoms. QRS remained lengthened at 140 msec. Another 100 mEq of sodium bicarbonate was administered, but the symptoms did not improve. In fact, the patient also began with increased irregular heartbeats at the ventricles. Because of this, further sodium bicarbonate was avoided, and the patient was instead given 1.5 cc/kg of a 20% intravenous lipid emulsion. No change occurred, but after twenty minutes, when the patient was given a second 1.5 cc/kg dose of the 20% lipid emulsion, within five minutes, his cardiac symptoms suddenly improved. His heart rate slowed, and his QRS shortened to 106 msec. The patient’s symptoms all began to resolve, and he was soon discharged [12]. 

Cherukuri and colleagues detail a case in their 2019 study of a 24-year-old female patient who ingested 360 tablets of 50 mg diphenhydramine each for a total ingestion of 18 g diphenhydramine. She initially presented with tonic–clonic seizure, then postictal state, low blood pressure, for which she was treated with sodium chloride and norepinephrine drip, and metabolic acidosis, for which she was treated with sodium bicarbonate. However, her symptoms progressed to a typical anticholinergic presentation, including comatose status, dilated pupils, high blood pressure, tachycardia with lengthened QRS and QTc intervals, and further seizures. The seizures resolved with lorazepam, but other symptoms persisted despite additional treatment with sodium chloride, sodium bicarbonate, and intravenous magnesium. Due to continued symptoms, the decision was made to try lipid emulsion therapy. The patient received a 1.5 mL/kg dose of 20% intravenous lipid emulsion, and then a 25 mL/h intravenous lipid emulsion infusion was given for 24 h. The QRS and QTc intervals soon shortened, and the patient’s blood pressure improved. By 48 h, the patient’s symptoms had greatly improved, and she was soon discharged without further complications [15].

These studies suggest that lipid emulsion therapy may successfully help treat diphenhydramine overdose. However, side effects of lipid emulsion therapy exist, which may include pancreatitis, interference in blood lipid labs, and even acute respiratory distress syndrome [12]. Additionally, not enough data exists yet to compare cases treated with lipid emulsion to those treated without in order to precisely determine the effectiveness of lipid emulsion therapy. Because of this, some authors have argued that lipid emulsion therapy should be considered an adjunctive therapy, to be used when other previously discussed treatment methods do not work or in combination with such existing treatments, especially in more severe diphenhydramine overdose cases [15]. Its potential utility as an alternative to sodium bicarbonate in addressing the adverse cardiac effects of severe diphenhydramine toxicity cases is not to be ignored. Future studies are needed to further elucidate the efficacy and safety of lipid emulsion therapy and to determine the situations in which its administration is most appropriate. 

## 3. Conclusions

**Figure 2:**
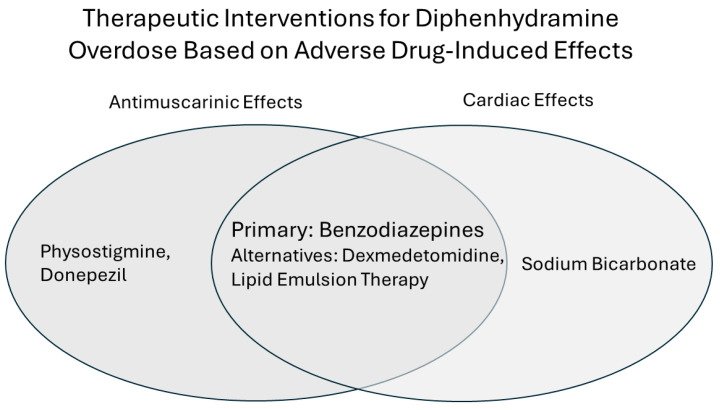
Summary comparing the theoretical therapies for diphenhydramine overdose with the traditional approach using a benzodiazepine such as Lorazepam.

The five different treatment methods detailed in this review, in addition to already existing standard benzodiazepine treatments, represent the current spectrum of therapies available for diphenhydramine overdose (see Figure 2). Recent research on physostigmine, sodium bicarbonate, donepezil, dexmedetomidine, and lipid emulsion therapy has proved promising so far, suggesting success in treating diphenhydramine toxicity without some of the drawbacks of previous therapies. 

Altogether, the treatments discussed here represent an arsenal of tools from which a healthcare team can draw to usher diphenhydramine overdose patients towards recovery. These treatments, whether used individually or in conjunction with one another, could help care teams address adverse symptoms in a diverse array of patient demographics and in overdose mono or poly ingestions. In light of diphenhydramine overdoses, a growing public health concern, further research into the safety of these and new treatments is needed more than ever. As efforts grow towards potentially restricting diphenhydramine usage and availability, the development of more robust treatments for diphenhydramine toxicity could help healthcare professionals effectively manage overdose cases in the meantime. 

## References

[1] Wolfson A.R., Wong D., Abrams E.M., Waserman S., Sussman G.L. (2022). Diphenhydramine: Time to Move on?. J. Allergy Clin. Immunol. Pract..

[2] Merenstein D., Diener-West M., Halbower A.C., Krist A., Rubin H.R. (2006). The trial of infant response to diphenhydramine: The TIRED study--a randomized, controlled, patient-oriented trial. Arch. Pediatr. Adolesc. Med..

[3] Sharma A.N., Hexdall A.H., Chang E.K., Nelson L.S., Hoffman R.S. (2003). Diphenhydramine-induced wide complex dysrhythmia responds to treatment with sodium bicarbonate. Am. J. Emerg. Med..

[4] Okuno T., Morimoto S., Nishikawa H., Haraguchi T., Kojima H., Tsujino H., Arisawa M., Yamashita T., Nishikawa J., Yoshida M. (2020). Bitterness-Suppressing Effect of Umami Dipeptides and Their Constituent Amino Acids on Diphenhydramine: Evaluation by Gustatory Sensation and Taste Sensor Testing. Chem. Pharm. Bull..

[5] Kuo C.C., Huang R.C., Lou B.S. (2000). Inhibition of Na^+^ current by diphenhydramine and other diphenyl compounds: Molecular determinants of selective binding to the inactivated channels. Mol. Pharmacol..

[6] Simons F.E., Simons K.J. (2011). Histamine and H1-antihistamines: Celebrating a century of progress. J. Allergy Clin. Immunol..

[7] FDA (2020). Benadryl (Diphenhydramine): Drug Safety Communication—Serious Problems with High Doses of the Allergy Medicine. https://www.fda.gov/safety/medical-product-safety-information/benadryl-diphenhydramine-drug-safety-communication-serious-problems-high-doses-allergy-medicine.

[8] Spector R., Choudhury A.K., Chiang C.K., Goldberg M.J., Ghoneim M.M. (1980). Diphenhydramine in Orientals and Caucasians. Clin. Pharmacol. Ther..

[9] Ten Eick A.P., Blumer J.L., Reed M.D. (2001). Safety of antihistamines in children. Drug Saf..

[10] Zekhtser M., Carroll E., Boyd M., Ambati S. (2021). The Role of Dexmedetomidine in Pediatric Patients Presenting with an Anticholinergic Toxidrome. Case Rep. Crit. Care.

[11] Walker A., Delle Donne A., Douglas E., Spicer K., Pluim T. (2014). Novel use of dexmedetomidine for the treatment of anticholinergic toxidrome. J. Med. Toxicol..

[12] Abdi A., Rose E., Levine M. (2014). Diphenhydramine overdose with intraventricular conduction delay treated with hypertonic sodium bicarbonate and i.v. lipid emulsion. West. J. Emerg. Med..

[13] Dinwiddie A.T., Tanz L.J., Bitting J. (2022). Notes from the Field: Antihistamine Positivity and Involvement in Drug Overdose Deaths—44 Jurisdictions, United States, 2019-2020. MMWR Morb. Mortal. Wkly. Rep..

[14] Padilla R.B., Pollack M.L. (2002). The use of physostigmine in diphenhydramine overdose. Am. J. Emerg. Med..

[15] Cherukuri S.V., Purvis A.W., Tosto S.T., Velayati A. (2019). IV Lipid Emulsion Infusion in the Treatment of Severe Diphenhydramine Overdose. Am. J. Case Rep..

[16] Burns M.J., Linden C.H., Graudins A., Brown R.M., Fletcher K.E. (2000). A comparison of physostigmine and benzodiazepines for the treatment of anticholinergic poisoning. Ann. Emerg. Med..

[17] Phillips M.A., Acquisto N.M., Gorodetsky R.M., Wiegand T.J. (2014). Use of a physostigmine continuous infusion for the treatment of severe and recurrent antimuscarinic toxicity in a mixed drug overdose. J. Med. Toxicol..

[18] Bruccoleri R.E., Burns M.M. (2016). A Literature Review of the Use of Sodium Bicarbonate for the Treatment of QRS Widening. J. Med. Toxicol..

[19] Cole J.B., Stellpflug S.J., Gross E.A., Smith S.W. (2011). Wide complex tachycardia in a pediatric diphenhydramine overdose treated with sodium bicarbonate. Pediatr. Emerg. Care.

[20] Ahmad J., Hasan M.J., Anam A.M., Barua D.K. (2019). Donepezil: An unusual therapy for acute diphenhydramine overdose. BMJ Case Rep..

[21] Rogers S.L., Friedhoff L.T. (1998). Pharmacokinetic and pharmacodynamic profile of donepezil HCl following single oral doses. Br. J. Clin. Pharmacol..

[22] Rogers S.L., Yamanishi Y., Yamatsu K., Becker R., Giacobini E. (1991). E2020—The Pharmacology of a Piperidine Cholinesterase Inhibitor. Cholinergic Basis for Alzheimer Therapy. Advances in Alzheimer Disease Therapy.

[23] Weinberg G.L. (2012). Lipid emulsion infusion: Resuscitation for local anesthetic and other drug overdose. Anesthesiology.

